# Selection of Internal Control Genes for Real-Time Quantitative PCR in Ovary and Uterus of Sows across Pregnancy

**DOI:** 10.1371/journal.pone.0066023

**Published:** 2013-06-13

**Authors:** María Martínez-Giner, José Luis Noguera, Ingrid Balcells, Amanda Fernández-Rodríguez, Ramona N. Pena

**Affiliations:** 1 IRTA, Genètica i Millora Animal, Lleida, Spain; 2 CRAG, Departament de Genètica Animal, Universitat Autònoma de Barcelona, Spain; 3 INIA, Mejora Genética Animal, Madrid, Spain; 4 Departament de Producció Animal, Universitat de Lleida, Lleida, Spain; Northwestern University, United States of America

## Abstract

**Background:**

Reproductive traits play a key role in pig production in order to reduce costs and increase economic returns. Among others, gene expression analyses represent a useful approach to study genetic mechanisms underlying reproductive traits in pigs. The application of reverse-transcription quantitative PCR requires the selection of appropriate reference genes, whose expression levels should not be affected by the experimental conditions, especially when comparing gene expression across different physiological stages.

**Results:**

The gene expression stability of ten potential reference genes was studied by three different methods (*geNorm*, *NormFinder* and *BestKeeper*) in ovary and uterus collected at five different physiological time points (heat, and 15, 30, 45 and 60 days of pregnancy). Although final ranking differed, the three algorithms gave very similar results. Thus, the most stable genes across time were *TBP and UBC* in uterus and *TBP* and *HPRT1* in ovary, while *HMBS* and *ACTB* showed the less stable expression in uterus and ovary, respectively. When studied as a systematic effect, the reproductive stage did not significantly affect the expression of the candidate reference genes except at 30d and 60d of pregnancy, when a general drop in expression was observed in ovary.

**Conclusions:**

Based in our results, we propose the use of *TBP*, *UBC* and *SDHA* in uterus and *TBP*, *GNB2L1* and *HPRT1* in ovary for normalization of longitudinal expression studies using quantitative PCR in sows.

## Introduction

Reverse-transcription quantitative PCR (qPCR) is a well-established method for estimating quantities of mRNA sequences. It has greater sensitivity than other quantitation methods [1] and application of relative quantitation protocols eliminate the need of standards with known target concentrations. In this type of study it is critical to normalize the amount of starting material with the use of one or more internal reference genes, which, ideally, should be present at constant levels across all samples in the experiment, at approximately equal concentration and amplified with equal efficiency as the target sequence [2]. Normalization through a reference gene adjusts for differences in the amount and quality of starting material and differences in RNA preparation and cDNA synthesis [3]. Still, the main drawback of relative quantitation remains in the selection of an appropriate reference gene, whose expression levels would not be affected by the experimental conditions. This problem is especially relevant when comparing expression across a wide spectrum of tissues or when studying one tissue at different physiological or developmental stages. The use of unconfirmed reference genes for normalization may drive to misleading interpretation of gene expression results [4]. For this reason it is critical to assess the reference genes before starting the quantitation experiment. The objective of the present study is to evaluate ten potential reference genes to be used as endogenous controls in time-course ovary and uterus gene expression analysis in sows at different reproductive stages and to compare three publicly available tools of reference gene selection.

## Results

### Gene Expression Profile Analyses at Different Physiological Stages

An initial evaluation of the expression profiles of the selected candidate reference genes indicated that all of them were expressed across all the physiological stages (heat, and 15, 30, 45 and 60 days of pregnancy), both in uterus and ovary of Meishan×Iberian F_2_ sows. Most genes gave Ct values in the range of 18–24 except for *HPRT1* and *HMBS*, which were less abundant than the rest (Table 1). Therefore, the ten genes were retained in the study and expression data was obtained for all candidate reference genes in all ovary (n = 32) and uterus (n = 30) cDNA samples (Table 2).

**Table 1:** Mean Ct values and gene stability values provided by *geNorm* (M) and *NormFinder* (Sv), and Pearson correlation coefficient (r_I_) with calculated index from *BestKeeper*.

Gene	Uterus				Ovary			
	Ct	*GeNorm*	*NormFinder*	*BestKeeper*	Ct	*GeNorm*	*NormFinder*	*BestKeeper*
		(M)	(Sv)	(r_I_)		(M)	(Sv)	(r_I_)
*ACTB*	18.35	1.271	0.510	0.860	20.77	1.198	0.499	0.955
*B2M*	18.69	1.578	0.541	0.565	18.82	0.897	0.384	0.878
*GNB2L1*	21.52	0.972	0.307	0.909	21.78	0.774	0.313	0.914
*HMBS*	27.23	1.597	0.673	0.463	27.47	0.866	0.351	0.874
*HPRT1*	26.54	1.088	0.275	0.778	26.03	**0.729**	**0.219**	0.957
*RPL32*	22.26	1.003	0.321	0.904	22.34	0.788	0.311	0.913
*SDHA*	24.61	0.965	0.276	0.895	23.98	0.869	0.368	0.889
*TBP*	24.65	**0.875**	**0.159**	**0.968**	24.91	**0.675**	**0.164**	**0.979**
*UBC*	20.75	**0.944**	0.309	**0.918**	21.33	0.907	0.457	0.881
*YWHAZ*	21.48	1.101	**0.263**	0.906	21.67	0.869	0.421	**0.990**

The two most stable genes in each analysis are indicated in bold.

**Table 2:** Distribution of samples used/collected from the 32 sows of this experiment.

	Heat	Pregnancy				TOTAL
		15d	30d	45d	60d	
**Ovary**	8/8	6/6	7/8	6/6	4/4	31/32
**Uterus**	7/7	6/6	6/8	6/6	3/3	28/30

The *BestKeeper* software recommends the exclusion of expression outliers as these can obscure the accuracy of gene expression estimation. An intrinsic variance (InVar) of expression for each individual sample is calculated as the deviance of this sample value in relation to the mean value of all samples in the same physiological stage and tissue. This high InVar could be due to inefficient reverse transcription (RT) reaction or cDNA degradation [5]. Based on this parameter, strongly deviating samples, over a 3-fold over or under expression, were removed. This affected one sample of ovary and two of uterus (Table 2) and the rest were retained for further gene stability analysis.

### Gene Expression Stability Analyses

**Figure 1:**
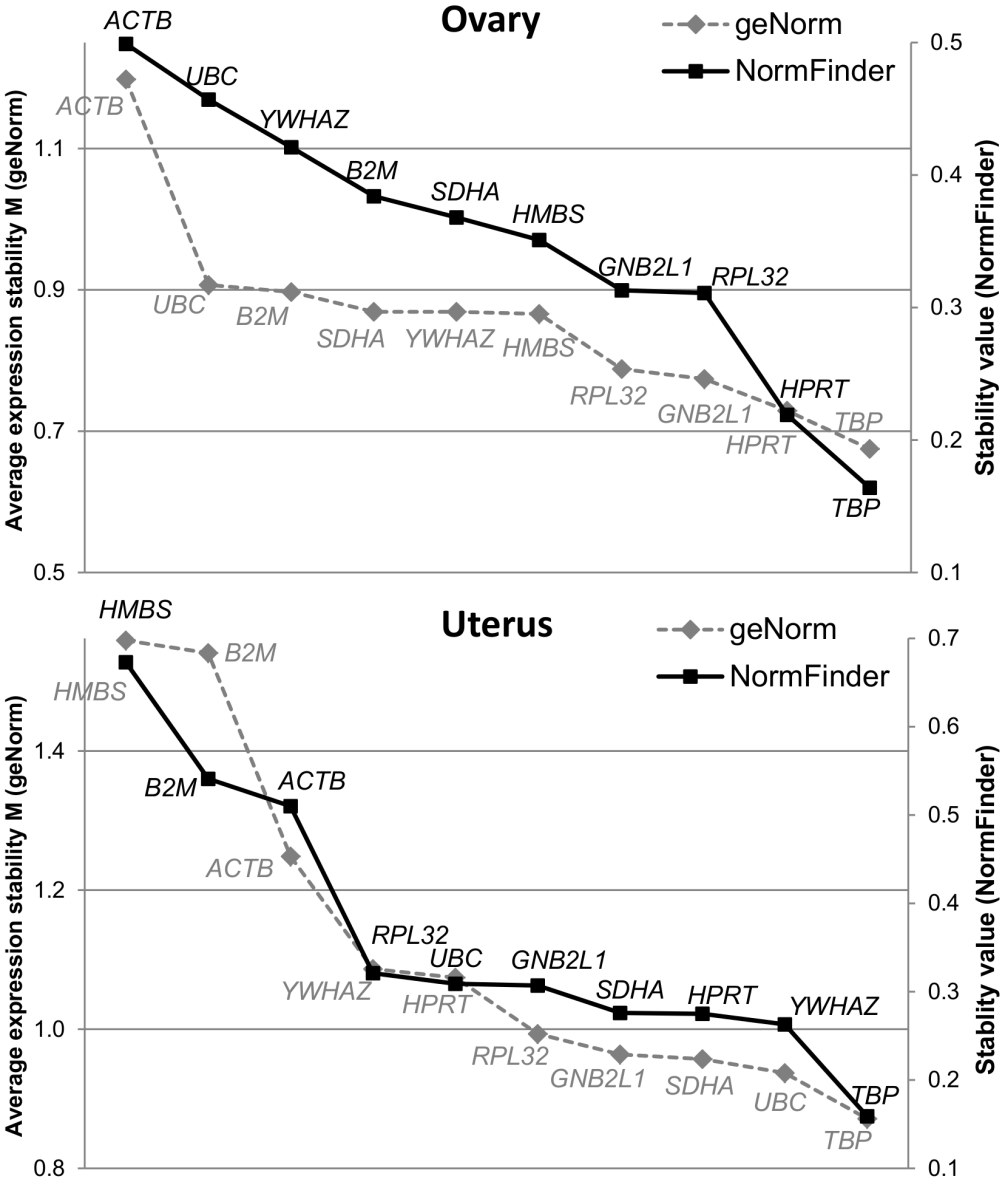
Rank of gene stability value provided by *geNorm* and *NormFinder.* Ten potential reference genes were tested for gene stability in uterus and ovary of 32 sows slaughtered at heat and four time points during pregnancy.

The gene expression stability study was performed with three publicly available tools that used self-developed statistical algorithms to analyze expression data. Analysis performed with the first of these tools, *geNorm* indicated that ovary and uterus differed in the most stable genes (Figure 1). Thus, although for both tissues the most stable gene across physiological stages was *TBP*, the second most stable gene was *UBC* for uterus and *HPRT1* for ovary. The other reference genes showed an intermediate stability in both tissues, except for *–HMBS* and *B2M*, which expression was very unstable in uterus. In this study, gene stability values (index *M*, see Materials and Methods) were judged acceptable if <1.5 as in [6], although this cut-off value is fully arbitrary and must be adapted to the singularities of each tissue and/or experiment. For instance, the same candidate reference genes analyzed within one single tissue type and under the same treatment or physiological conditions are prone to give lower *M* values than in a study across tissues and/or across conditions. In our case, *M* ranged from 0.875 to 1.597 in uterus and from 0.675 to 1.198 in ovary, which would indicate an overall major stability in gene expression in ovary across times (Figure 1 and Table 1). These results reinforce the need to adjust for appropriate endogenous controls in each experiment.

Results with *NormFinder* showed overall the same stability trend (*Sv* value in Table 1) in gene expression in uterus and ovary as with *geNorm*, although a narrower range in expression stability value was obtained (0.159 to 0.673 in uterus and 0.164 to 0.499 in ovary; see Figure 1 and Table 1). Nevertheless, there were some minor differences in ranking when compared to *geNorm* results. For instance, in uterus the second most stable gene was *YWHAZ* (*Sv* = 0.263) instead of *UBC* (*Sv* = 0.309). In ovary, the most stable gene was *TBP* (*Sv* = 0.164), being *HPRT1* (*Sv* = 0.219) in the second place.

Uterus and ovary displayed different profiles of gene stability as assessed both with geNorm (*M*) and NormFinder (*Sv*) (Figure 1). In uterus, most expression instability was due to three genes (*HMBS*, *B2M* and *ACTB*), while the remaining seven genes exhibited more similar stability values (Figure 1). In contrast, gene stability was more uneven in ovary, with more gradual differences between genes.

One advantage of *NormFinder* with regard to the two other tools is that it can estimate the intra-group gene expression variation for each individual gene. In our case, groups were defined as the different reproductive stages of the sows for each tissue. Results showed an overall higher variation in uterus in all the genes, with intra-group variation ranged between less than 0.001 and 3.141 (Table S1). In ovary, intra-group variation was lower (0.001 to 0.470) except for *ACTB* gene (0.053 to 1.422), which was the least stable gene in ovary. Together, geNorm and NormFinder results indicate that expression of nine candidate genes in uterus (*M*>1.5 for *ACTB*) and the ten of them in ovary is rather stable within the physiological stages analyzed.

Finally, *BestKeeper* estimates Pearson correlations between the expression levels of all reference genes, and combine all the highly correlated genes into a Variation Index, defined as the geometric mean of expression value of all the contributing reference genes. Pearson correlation coefficient (r) between expression values of the ten reference gene in ovary was high (Table S2) and ranged from 0.644 (between *GNB2L1* and *SDHA*) to 0.993 (between *RPL32* and GNB2L1). In uterus, *B2M* and *HMBS* systematically showed lower correlation values with all the other genes, being the correlation between the two genes 0.082. However, the correlation coefficient between the other eight genes was high: ranging from 0.572 between *HPRT1* and *ACTB* to 0.92 between *GNB2L1* and *ACTB* (Table S2). For both tissues, the highest stability index was achieved by *TBP*, followed by *UBC* in uterus, and by *YWHAZ* in ovary (Table 1).

**Figure 2:**
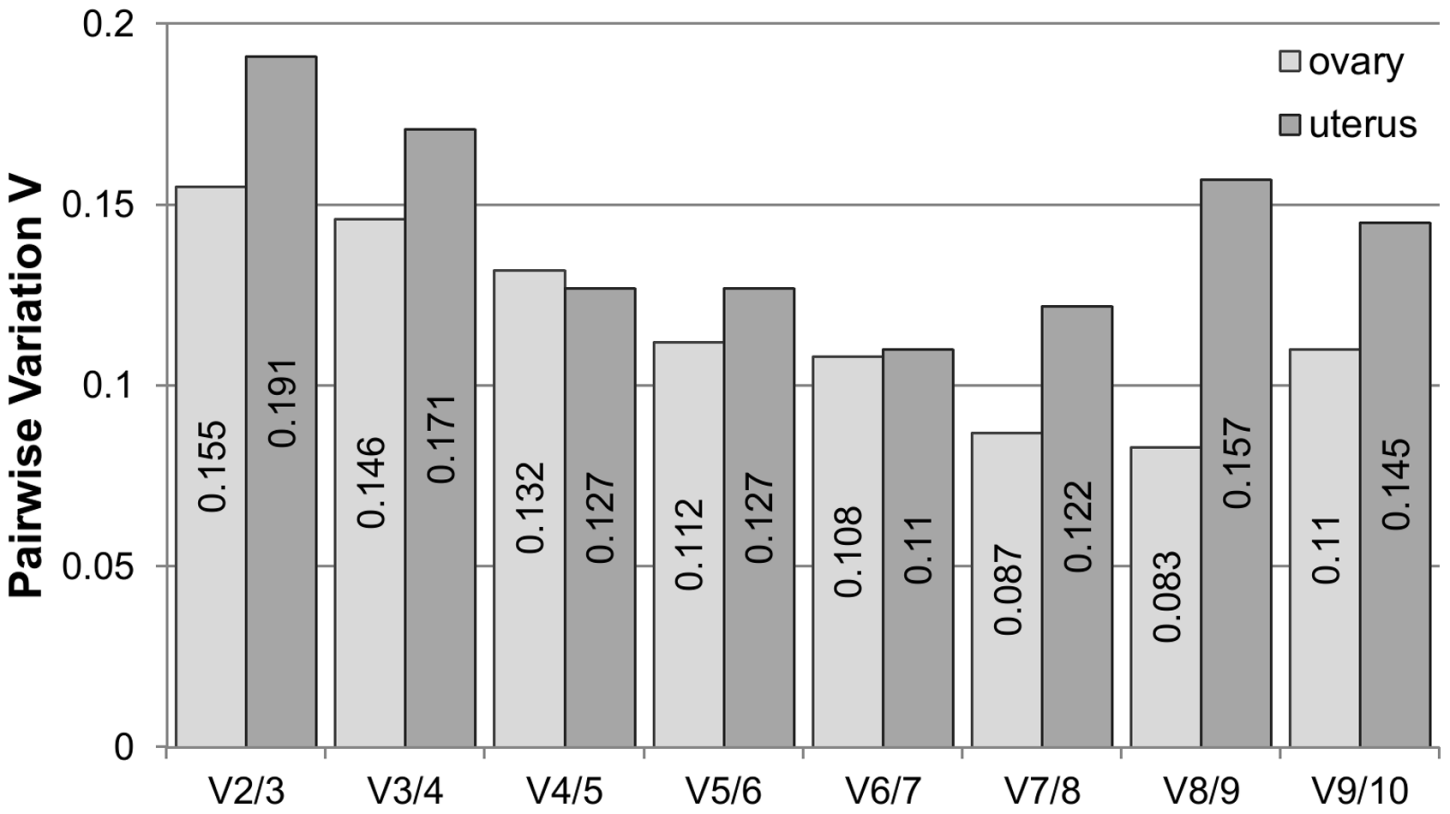
*geNorm* estimation of pairwise variation of gene expression. Changes in estimation of gene expression by using increasing number of the ten potential reference genes in uterus and ovary as provided by *geNorm*.

A normalization factor study was performed with *geNorm* to evaluate the optimum number of reference genes for real-time qPCR in our experimental conditions (Figure 2). Genes were added following increasing *M* values as indicated in Table 1. The pairwise variation (*V*) between two sequential normalization factors with an increasing number of genes was above 0.15 in ovary (between 0.110 and 0.191) while the uterus was close to this threshold (0.083 to 0.155. Although a *V* of 0.15 was proposed by Vandesompele *et al.* [6] as cut-off value below which the inclusion of an additional control gene is not required, this value is also arbitrary and must be adjusted to each experiment. In uterus, increasing the number of reference genes from 3 to 10 did not improve the pairwise variation (Figure 2) and, therefore, adding those less stable genes would not advantageous. Similarly, although the pairwise variation value (*V*) is lower in ovary, inclusion of up to ten genes does not reduce *V* (Figure 2). All together, these results indicate that the optimum number of the reference genes that should be included in a gene expression study would be four for uterus and three in ovary.

**Figure 3:**
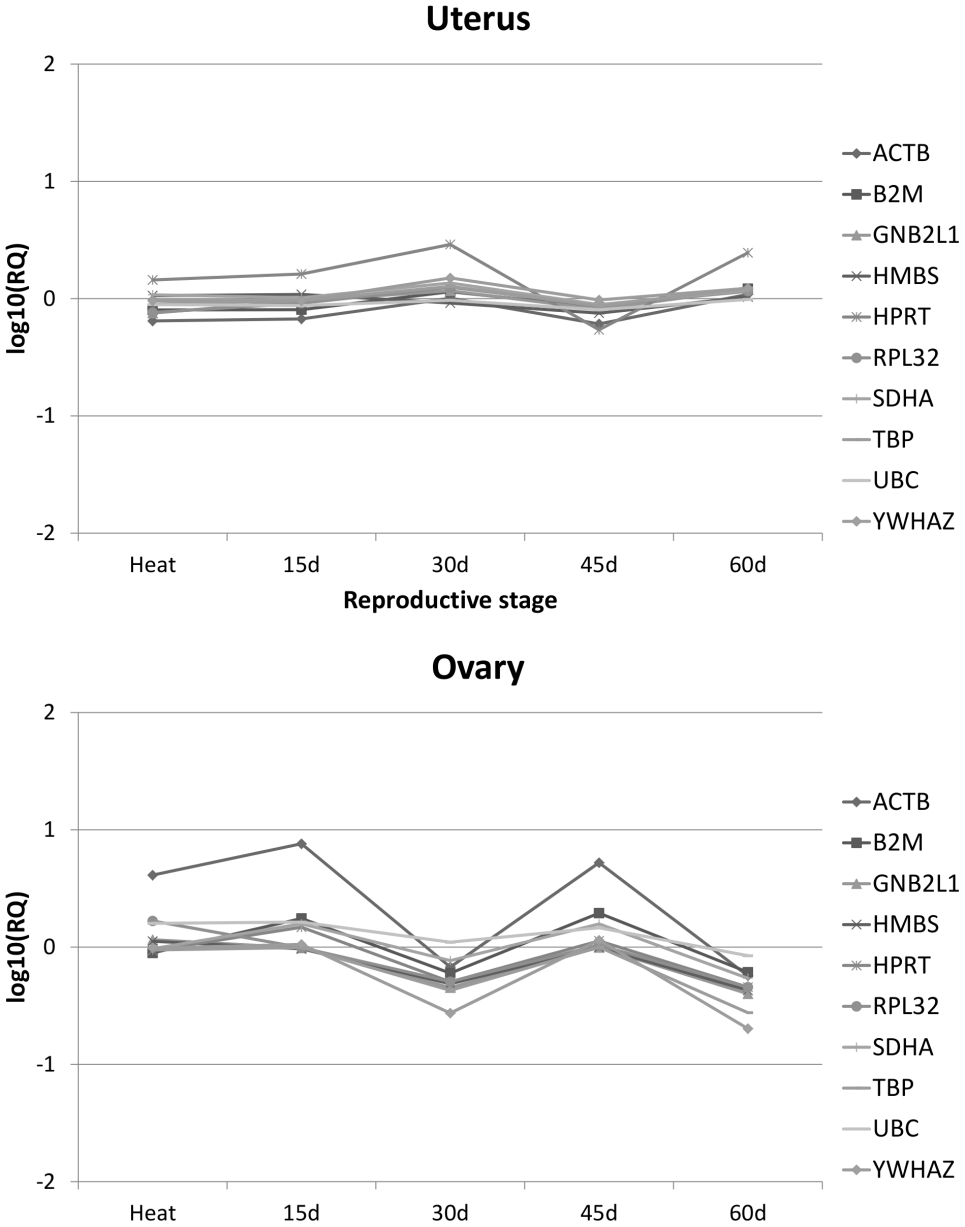
Relative quantitation of the ten potential reference genes in ovary and uterus. Log_10_-transformed relative quantitation levels are shown across the different physiological stages.

To further analyze the effect of the reproductive stage of the animal in the expression of the ten potential reference genes, we applied a simple Bayesian statistical model (Table 3). Results indicate that the reproductive stage of the sow has an effect in the expression of most reference genes at 30 and 60 days of pregnancy in ovary, while expression in uterus only was affected by reproductive stages at some isolated time points (Table 3). Overall, there is a lower expression level in seven (30 days of pregnancy) and nine (60 days of pregnancy) of the ten genes tested in ovary (Table 3 and Figure 3).

**Table 3:** Posterior mean (PM) and posterior standard deviation (PSD) of expression level in logarithmic scale of each potential reference gene in each physiological stage evaluated in this study for uterus and ovary.

Genes		Uterus					Ovary				
		Heat	15 days	30 days	45 days	60 days	Heat	15 days	30 days	45 days	60 days
*ACTB*	PM	−0.19^a^	−0.17^a^	0.00^a^	−0.21^a^	0.03^a^	0.61^a^	0.87^a^	−0.16^b^	0.71^a^	−0.24^b^
	PSD	[−0.28, −0.07]	[−0.27, −0.06]	[−0.11,0.12]	[−0.40,0.01]	[−0.08,0.16]	[0.26,0.90]	[0.70,1.06]	[−0.18,0.24]	[0.40,1.04]	[−0.44, −0.05]
*B2M*	PM	−0.09^ab^	−0.09^a^	0.05^b^	−0.07^ab^	0.08^b^	−0.04^a^	0.24^b^	−0.21^a^	0.28^b^	−0.21^a^
	PSD	[−0.24, −0.01]	[−0.14, −0.05]	[−0.04,0.16]	[−0.16,0.01]	[−0.04,0.19]	[−0.16,0.10]	[0.11,0.40]	[−0.34, −0.10]	[0.14,0.43]	[−0.36, −0.05]
*GNB2L1*	PM	−0.12^a^	−0.01^ab^	0.06^b^	−0.09^ab^	0.08^b^	0.06^a^	0.00^a^	−0.34^b^	0.00^a^	−0.39^b^
	PSD	[−0.19, −0.06]	[−0.08,0.05]	[−0.00,0.12]	[−0.17,0.01]	[−0.01,0.17]	[−0.06,0.19]	[−0.16,0.14]	[−0.48, −0.20]	[−0.16,0.14]	[−0.57, −0.23]
*HMBS*	PM	0.02^a^	0.03^a^	−0.03^a^	−0.12^a^	0.01^a^	0.04^a^	−0.02^a^	−0.31^b^	0.00^a^	−0.36^b^
	PSD	[−0.06,0.14]	[−0.08,0.12]	[−0.13,0.06]	[−0.27, −0.01]	[−0.15,0.14]	[−0.04,0.14]	[−0.13,0.10]	[−0.42, −0.19]	[−0.11,0.11]	[−0.53, −0.23]
*HPRT1*	PM	0.15^ab^	0.20^a^	0.46^a^	−0.26^b^	0.39^a^	−0.02^ab^	0.17^a^	−0.29^b^	0.05^a^	−0.34^b^
	PSD	[−0.04,0.38]	[0.04,0.41]	[0.04,0.91]	[−0.60,−0.01]	[0.09,0.74]	[−0.18,0.11]	[0.01,0.34]	[−0.44, −0.14]	[−0.09,0.22]	[−0.56, −0.13]
*RPL32*	PM	−0.11^a^	−0.03^ab^	0.09^b^	−0.05^ab^	0.06^ab^	0.21^a^	−0.01^a^	−0.29^b^	0.01^a^	−0.34^b^
	PSD	[−0.24, −0.04]	[−0.15,0.06]	[−0.01,0.18]	[−0.15,0.02]	[−0.07,0.19]	[0.10,0.34]	[−0.13,0.16]	[−0.40, −0.15]	[−0.09,0.16]	[−0.50, −0.18]
*SDHA*	PM	0.03^ab^	0.01^ab^	0.13^a^	−0.05^b^	0.06^ab^	−0.01^ab^	0.20^a^	−0.11^ab^	0.19^a^	−0.26^b^
	PSD	[−0.06,0.09]	[−0.07,0.09]	[0.05,0.20]	[−0.12,0.01]	[−0.03,0.17]	[−0.17,0.14]	[0.02,0.38]	[−0.25,0.05]	[0.02,0.36]	[−0.48, −0.05]
*TBP*	PM	−0.04^a^	−0.01^a^	0.10^a^	−0.06^a^	0.09^a^	−0.02^a^	0.00^a^	−0.36^b^	0.00^a^	−0.55^b^
	PSD	[−0.11,0.05]	[−0.10,0.05]	[0.02,0.18]	[−0.17,0.03]	[−0.04,0.20]	[−0.16,0.11]	[−0.15,0.14]	[−0.50, −0.22]	[−0.16,0.15]	[−0.74, −0.36]
*UBC*	PM	−0.05^a^	−0.05^a^	−0.01^a^	−0.09^a^	−0.01^a^	0.20^a^	0.21^a^	0.03^ab^	0.16^ab^	−0.07^b^
	PSD	[−0.10,0.01]	[−0.10, −0.01]	[−0.05,0.02]	[−0.14, −0.04]	[−0.05,0.03]	[0.10,0.30]	[0.08,0.33]	[−0.07,0.14]	[0.03,0.28]	[−0.21,0.06]
*YWHAZ*	PM	−0.01^a^	−0.01^a^	0.17^a^	−0.01^a^	0.08^a^	0.00^a^	0.02^a^	−0.56^b^	0.05^a^	−0.69^b^
	PSD	[−0.19,0.07]	[−0.13,0.08]	[0.04,0.28]	[−0.13,0.08]	[−0.03,0.22]	[−0.17,0.17]	[−0.18,0.24]	[−0.75, −0.38]	[−0.16,0.27]	[−0.92,−0.45]

a,b Different index within the same row indicate the posterior distributions of the difference was over zero with a probability lower than 0.05.

## Discussion

Reference genes in gene expression studies should ideally show a stable level of expression across cell types or biological state of the tissue. Moreover, it should not be regulated by the experimental conditions, such as rearing environment, external treatments or development stages or physiological state. This is particularly important when time-course experiments are analyzed in metabolically active tissues such as the mammary gland [7]. Ovary and uterus also undergo substantial physiological changes along ovarian cycle and pregnancy. For instance, hormone production in the ovary switches from estrogens at heat to progesterone by the luteal bodies during gestation. The uterus also undergoes physiological and developmental changes during pregnancy. For instance, in sows the maternal recognition of gestation takes place around the 12^th^–15^th^ day. Fetal implantation finishes at day 28–30 and 65% of prenatal mortality happens at this stage. The remaining prenatal mortality occurs mostly before day 40–45, so the number of implanted fetus at day 45 and, of course, at day 60, is a good indicator of litter size [8].

Probably inherited from *northern blot analysis*, *ACTB*, *HPRT1* and *GAPDH* have been widely used as reference genes for qPCR analysis, despite the enormous body of evidence indicating that their transcription levels are not constant across many developmental stages and experimental conditions [9], [10]. To overcome this limitation, several tools have been made available to assess the stability of expression of candidate reference genes for qPCR gene expression analysis [5], [6], [11]. Most of these studies have focused on a specific tissue type [9], [12]–[14]. The disparity of valid reference genes obtained in each study stresses the importance of selecting suitable controls for each experiment. In pigs, there has been a valuable contribution from a number of authors describing suitable reference gene in time-course study of early embryonic development [12], with-in tissue in seven tissue types [13], and across-tissues [9] from a panel of 17 different tissues. A recent study has focused on sow endometrium at 12 days of pregnancy [14] and reference sequences for small-non coding RNA studies (such as miRNA) have also recently been evaluated in the uterus of 20 days-pregnant sows [15]. In contrast, there are no published reports describing qPCR reference genes for pig ovary studies. Moreover, the stability of reference genes across different reproductive stages has not been evaluated in pig in uterus nor in ovary.

Since there is no consensus about which is the most accurate algorithm to estimate gene expression stability, the comparison of the different methods could be the best way to identify the best reference genes to be used. We compared three different statistical approaches (*geNorm*, *NormFinder* and *BestKeeper*) to evaluate our ten potential reference genes. The three algorithm use the Ct values provided by the real time PCR data acquisition software, as they are normal distributed and parametric test can be performed in correlation and regression analysis. However, *BestKeeper* relies on repeated pair-wise correlation analysis of expression levels to elaborate an index that measures the relevance of the contribution of each reference gene to the mean Ct values for each experimental sample set. Pearson correlation coefficient used by *BestKeeper* is invalidated if the reference genes display heterogeneous variances. In this scenario, Spearman correlation is more adequate but the algorithm is yet to be implemented with this option. To overcome this limitation, the authors recommend avoiding low expressed genes (giving Ct values of around 30–35 cycle) trying to use genes giving similar Ct data [5]. In contrast, the gene stability measure (*M*) developed within the *GeNorm* control selection tool relies on the principle that the expression ratio of two ideal internal control genes is identical in all samples and is not affected by the experimental conditions. Assuming that finding a gene that is constantly expressed in all possible situations is virtually impossible, *M* gives an idea of how much the expression of one of the genes changes with respect to the other. Higher *M* values correspond to increasing variation in expression ratio and, therefore, in lower gene expression stability. *NormFinder*, the third algorithm for reference gene selection evaluates the expression stability of each single reference gene and takes into account intra- and intergroup variations as deviation from the mean of the sample set to compute a stability value (*Sv*) which accounts for the error generated in the model for including that particular candidate reference gene.

One of the limitations of these algorithms is that decisions are made based on ranks of gene expression stability indexes, but none of the tools tested analyzes whether the experimental conditions (tissue, time, treatments, etc) significantly affect the gene expression values. We have explored this by means of a simple statistical model which included the reproductive state of the animals as a systematic effect. Many factors introduce errors to affect the accuracy and precision of quantitative analyses. Systematic errors introduce a constant bias into the results and, unlike random errors, which can be reduced by repeated measurements, cannot be detected by statistical means. Our results indicate that the expression of the ten candidate reference gene tested is overall not affected by the reproductive state of the animal in uterus, with some exception at isolated time points (*B2M*, *GNB2L1*, *HPRT1*, *RPL32*, *SDHA*). In contrast, there was a general drop in expression at day 30 and 60 of pregnancy which affected most genes in ovary (Figure 3 and Table 3). This does not seem to be due to the number of samples (lower, for instance, at 60 d) as these effects were not seen in uterus with similar number of samples tested. Instead, the effect of the reproductive stage over ovary must reflect the drastic changes in gene expression that take place in this organ at these time points. We believe this does not represent a weakness in our analysis; on the contrary, it can be taken as a strength as detection of systematic errors allows accounting for them in the model thus correcting the accuracy of the measurement.

Despite the diversity of mathematical approaches used by these selection tools, results regarding best reference genes for ovary and uterus samples across reproductive stages were very similar with the three methods. In ovary, *TBP* was the gene with higher stability while *ACTB* showed the worst stability. The expression of the ten genes in uterus resulted less stable than in ovary, as indicated by larger *M* values (*geNorm*), which is associated with more variable patterns of gene expression. Thus, in uterus, the most stable genes were *TBP* and *UBC*, while, for the three methods used, *HMBS* and *B2M* appeared as the worst options. In spite of this, there were some differences in the ranking in intermediate positions. The current practice in most qPCR experiments is to include several reference genes as it has been shown that normalizing with a single gene might lead to large errors. In this sense, Vandesompele *et al.* [6] suggested the use of the geometric mean between several validated reference genes to generate a normalization factor.

Our results indicate that the optimum number of reference genes for this geometric mean is four for uterus and three for ovary. Choosing the best three reference genes is recommend by Vandesompele *et al.* [6] and by Pfaffl *et al.* [5] for a more robust normalization of real-time PCR data. Also, from a practical point of view, three reference genes can be detected using fluorescent probes in a single reaction in most qPCR systems with four or five detection channels. In this context and taking into account the results obtained with the three algorithm tested the three selected genes would be *TBP*, *UBC* and *SDHA* for uterus and *TBP*, *GNB2L1* and *HPRT1* (*geNorm* and *NormFinder*) or *YWHAZ* (*BestKeeper*) for ovary.

### Conclusions

The analysis of these ten genes in time-course study of ovary and uterus of sows showed that *TBP*, *UBC* and *SDHA* were stable enough in uterus to be confidently used as reference genes for normalization of qPCR-based expression studies in this tissue, while *TBP*, *GNB2L1* and *HPRT1* would be reference genes of choice in time-course studies involving sow’s ovary.

## Methods

### Animal Material

A total of 32 multiparous (four parities or more) *Meishan* x *Iberian* F_2_ sows [16], [17] were slaughtered at different reproductive stages (heat, and 15, 30, 45 and 60 days of pregnancy, considering the day of the insemination as day 0) distributed as indicated in Table 2. Whole ovaries and samples from the apical uterus were collected, snap frozen in liquid nitrogen and stored at −80° until analysis. All animals were obtained according to the Spanish and European animal experimentation ethics law and approved by the Ethical and Care Committee of the institution (*Institut de Recerca i Tecnologies Agroalimentàries* – IRTA).

### RNA Isolation and cDNA Synthesis

Total RNA was isolated from each individual sample using the *FastRNA®Pro Green kit* (Qbiogene, Irvine, USA) according to the manufacturer’s instructions. RNA purity and concentration was evaluated with *Nanodrop ND-1000* spectrophotometer and verified in a 2100 *Bioanalyzer* (Agilent, Santa Clara, USA) obtaining a RIN number mean of 8.12 (range 7.30–9.00), which confirmed the good integrity of the starting material. The electrophoresis profiles were used to estimate the RNA integrity number (RIN). Reverse transcription to cDNA was performed from 1 µg of DNase-treated RNA with 100U of Revert-Aid retrotranscriptase (Fermentas, Burlington, Canada) and 0.2 µg of random hexamers using standard protocols [2] [1] [1] ^1^. Upon completion of reaction, cDNA samples were diluted 1∶10 with H_2_O prior to expression analysis.

### Selection of Reference Genes and Quantitative Real-time PCR

Ten potential reference genes were chosen for being frequently used as endogenous controls in expression studies by different authors [6], [9], [18]: beta-actin (*ACTB*), beta-2 microglobulin (*B2M*), guanine nucleotide binding protein (G protein), beta polypeptide 2-like 1 (*GNB2L1*), hydroxymethylbilane synthase (*HMBS*), hypoxanthine phosphoribosyltransferase 1 (*HPRT*), ribosomal protein L32 (*RPL32*), succinate dehydrogenase complex, subunit A, flavoprotein (Fp) (*SDHA*), TATA box binding protein (*TBP*), ubiquitin C (*UBC*), tyrosine 3-monooxygenase/tryptophan 5-monooxygenase activation protein, zeta polypeptide (*YWHAZ*). For each gene, a set of primers was designed with Primer Express Software v2.0 (Applied Biosystems, Foster City, USA) to quantitate the level of expression by real-time qPCR (Table 4). Primer pairs were designed so as to fall in different exons (as inferred from human or pig gene organization data) and to amplify a fragment of less than 250bp (Table 4). Fleige and Pfaffl [18] demonstrated that real-time qPCR based on short amplicons (70–250 bp) was independent of RNA integrity and therefore give more accurate results than long amplicons. Real time PCR reactions were performed in triplicate for each individual sample in a final volume of 5 µl containing 1x SYBRgreen PCR Master Mix (Applied Biosystems), 100 nM each primer and 1 µl of 1∶10 diluted cDNA on an ABI 7500 Real Time PCR System (Applied Biosystems). As a calibrator we used a pool of all RNA samples in the experiment. Data normalization and analysis were performed by the E^−ΔCt^ method [19] using as a calibrator a pool of all RNA samples of both tissues used in this study. PCR efficiency (E) was calculated as follow:
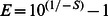
where S is the slope from the standard curve [19]. A dissociation curve analysis evidenced a single peak for all reactions indicating the specificity of the amplification and the absence of primer dimer formation. Ten-fold serial dilution of a cDNA template (generated from a mix of all RNA samples involved in this experiment) showed for all candidate reference genes an average amplification efficiency of 93.19% and an average correlation coefficient (R^2^) of 0.99 (Figure S1).

**Table 4:** Primers used in the real time quantitative PCR assay.

Primer Name	Sequence 5′→3′	NCBI Sequence	Exons	Size
ACTB_F	CACGCCATCCTGCGTCTGGA	XM_003357928.1	4,5	100 bp
ACTB_R	AGCACCGTGTTGGCGTAGAG			
B2M_F	TCGGGCTGCTCTCACTGTCT	NM_213978	1, 2	69 bp
B2M_R	GGCGTGAGTAAACCTGAACCTT			
GNB2L1_F	CCCGAGATAAAACCATCAAGCT	NM_214332	4, 5	93 bp
GNB2L1_R	CGGACACAAGACACCCACTCT			
HMBS_F	AGGATGGGCAACTCTACCTG	NM_001097412.1	15,16	83 bp
HMBS_R	GATGGTGGCCTGCATAGTCT			
HPRT1_F	AAGATGGTCAAGGTTGCAAGCT	NM_001032376.2	7, 8, 9	82 bp
HPRT1_R	ATTTCAAATCCAACAAAGTCTGGTCTA			
RPL32_F	CACCAGTCAGACCGATATGTCAA	NM_001001636	1, 2	70 bp
RPL32_R	CGCACCCTGTTGTCAATGC			
SDHA_F	CTACAAGGGGCAGGTTCTGA	XM_003362140.1	19, 20	141 bp
SDHA_R	AAGACAACGAGGTCCAGGAG			
TBP_F	AACAGTTCAGTAGTTATGAGCCAGA	DQ178129.1	8, 9, 10	153 bp
TBP_R	AGATGTTCTCAAACGCTTCG			
UBC_F	GCATTGTTGGCGGTTTCG	NM_001105309.1	2,3	64 bp
UBC_R	AGACGCTGTGAAGCCAATCA			
YWHAZ_F	TGATGATAAGAAAGGGATTGTGG	XM_001927228.4	3,4	134 bp
YWHAZ_R	CTCATAATAGAACACAGAGA			

### Analysis of Gene Expression Stability

Gene expression stability was evaluated with three different statistical algorithms: *BestKeeper* [5], *geNorm* [6] and *NormFinder* [11]. The three different software packages make use of the Ct values to determine the most stably expressed genes. *BestKeeper* analyzes the inter-gene relationship, calculating the Pearson correlation coefficient (r), the probability and the sample integrity and expression stability within each reference gene with an intrinsic variance of expression [5]. Data from the genes showing higher correlation values is combined to computes the geometric mean of Ct values (BestKeeper Index). Next, the Pearson’s correlation coefficient between each candidate reference gene and the index (r_I_) is calculated, which gives and estimation of the contribution of the gene to the BestKeeper Index. *GeNorm* determines the pairwise variation of a particular gene with all other control genes as the standard deviation of the logarithmically transformed expression ratios. A measure of internal control gene-stability (*M)* is defined by *GeNorm* as the average of the pairwise variation of one gene with all the other potential reference genes [6]. The lowest the *M* value, the more stable the expression of that gene is. To select the best-performing reference genes, the program recalculates the M stability measures after removal of the least stable gene and repeats the process until only the two most stable genes remain [6]. To test the minimum number of reference gene needed for adequate data normalization, *geNorm* calculates a pairwise variation (*V*) between using n and n+1 reference genes. Large *V* values indicate a significant effect of the additional gene on data normalization and endorse the need of including this gene among the controls. On the other hand, *NormFinder* [11] is a model-based approach that enables estimation not only of the overall variation of the candidate normalization genes, but also of the variation between subgroups of the same sample set. *NormFinder* combines the intra- and intergroup variation to estimate, for each individual gene, a stability value (*Sv*), which represents a practical measure of the systematic error that will be introduced when using the investigated gene. Candidate reference genes can then be ranked according to the *Sv* value, where lowest values correspond to the most stable genes.

### Statistical Association Analysis

In addition, an association analysis was performed with data from these genes. The following model was separately solved for each gene and tissue:
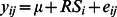
where:

**y_ij_** is the vector containing the log_10_-transformed expression data.

**μ** is the overall mean of each individual gene.

**R** is a vector with the systematic effect of the reproductive state of the sow (heat, 15, 30, 45 or 60 days of pregnancy).

**S_i_** is the incidence matrix for the systematic effect.

**e_ij_** is the vector of residual effects.

Expression data was transformed to the log_10_ scale to make data distribution more symmetric, attributing equal weight to conditions with overexpression or underexpression. This model was intended to evaluate the effect of the reproductive state on the level of expression of each candidate reference gene.

The model was solved by Bayesian inference. Assuming normality of the transformed expression values, the likelihood function of data is:
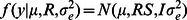



The prior distributions for μ, S, σ_e_, σ_μ_, were bounded uniform distributions. From the likelihood and prior distributions, the joint posterior distribution was developed for the unknown parameters of the model. The marginal posterior distributions of the unknown parameters were obtained using Gibbs Sampling [20]. The conditional distributions needed on the Gibbs sampler were Gaussian (μ, S) and inverted Chi Square (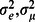). For each analysis, a total of 10,000 Gibbs iterations were performed after discarding the first 500. Convergence was checked using the Raftery and Lewis algorithm [21]. The statistical relevance of the reproductive state effect (k) was calculated from the distribution of the posterior standard deviation (PSD), by computing the probability (P) of these effects to be different from zero. Thus, we consider an effect as relevant when P over (when k>0) or below (when k<0) zero was bigger than 0.95.

## Supporting Information

Figure S1 **Linearity test and estimation of amplification efficiency for the PCR reaction of the ten candidate reference genes tested.**
(TIF)

Table S1 **Intra-group gene expression variation for each individual gene in each different reproductive stage for each tissue, calculated by** ***NormFinder*** **.**
(DOCX)

Table S2 **Pearson correlation coefficient (r_I_) between expression values of the ten reference gene in each tissue.**
(DOCX)

## References

[1] VanGuilder HD, Vrana KE, Freeman WM (2008) Twenty-five years of quantitative PCR for gene expression analysis. BioTechniques 44: 619–626. 18474036 10.2144/000112776

[2] Sambrook J, Russell D (2001) Molecular Cloning: A Laboratory Manual Cold Spring Harbor: Cold Spring Harbor Laboratory Press.

[3] Huggett J, Dheda K, Bustin S, Zumla A (2005) Real-time RT-PCR normalisation; strategies and considerations. Genes Immun 6: 279–284. 15815687 10.1038/sj.gene.6364190

[4] Haller F, Kulle B, Schwager S, Gunawan B, Heydebreck A, et al (2004) Equivalence test in quantitative reverse transcription polymerase chain reaction: Confirmation of reference genes suitable for normalization. Anal Biochem 335: 1–9. 15519565 10.1016/j.ab.2004.08.024

[5] Pfaffl MW, Tichopad A, Prgomet C, Neuvians TP (2004) Determination of stable housekeeping genes, differentially regulated target genes and sample integrity: BestKeeper–Excel-based tool using pair-wise correlations. Biotechnol Lett 26: 509–515. 15127793 10.1023/b:bile.0000019559.84305.47

[6] Vandesompele J, De Preter K, Pattyn F, Poppe B, Van Roy N, et al (2002) Accurate normalization of real-time quantitative RT-PCR data by geometric averaging of multiple internal control genes. Genome Biol 3: research0034.1–0034.11. 12184808 10.1186/gb-2002-3-7-research0034 PMC126239

[7] Tramontana S, Bionaz M, Sharma A, Graugnard D, Cutler E, et al (2008) Internal controls for quantitative polymerase chain reaction of swine mammary glands during pregnancy and lactation. J Dairy Sci 91: 3057–3066. 18650282 10.3168/jds.2008-1164

[8] Hafez B, Hafez ESE (2000) Reproduction in farm animals. Philadelphia: Wiley-Blackwell.

[9] Nygard AB, Jørgensen CB, Cirera S, Fredholm M (2007) Selection of reference genes for gene expression studies in pig tissues using SYBR green qPCR. BMC Mol Biol 8: 67. 17697375 10.1186/1471-2199-8-67 PMC2000887

[10] Robert C, McGraw S, Massicotte L, Pravetoni M, Gandolfi F, et al (2002) Quantification of housekeeping transcript levels during the development of bovine preimplantation embryos. Biol Reprod 67: 1465–1472. 12390877 10.1095/biolreprod.102.006320

[11] Andersen CL, Jensen JL, Ørntoft TF (2004) Normalization of real-time quantitative reverse transcription-PCR data: A model-based variance estimation approach to identify genes suited for normalization, applied to bladder and colon cancer data sets. Cancer Res 64: 5245–5250. 15289330 10.1158/0008-5472.CAN-04-0496

[12] Kuijk EW, Du Puy L, Van Tol HTA, Haagsman HP, Colenbrander B, et al (2007) Validation of reference genes for quantitative RT-PCR studies in porcine oocytes and preimplantation embryos. BMC Dev Biol 7: 58. 17540017 10.1186/1471-213X-7-58 PMC1896162

[13] Svobodová K, Bílek K, Knoll A (2008) Verification of reference genes for relative quantification of gene expression by real-time reverse transcription PCR in the pig. J Appl Genet 49: 263–265. 18670063 10.1007/BF03195623

[14] Wang S, Li J, Zhang A, Liu M, Zhang H (2011) Selection of reference genes for studies of porcine endometrial gene expression on gestational day 12. Biochem Bioph Res Co 408: 265–268. 10.1016/j.bbrc.2011.04.010 21501585

[15] Wessels JM, Edwards AK, Zettler C, Tayade C (2011) Selection and validation of reference genes for miRNA expression studies during porcine pregnancy. PloS One 6: e28940. 22174931 10.1371/journal.pone.0028940 PMC3236229

[16] Rodriguez C, Tomas A, Alves E, Ramirez O, Arque M, et al (2005) QTL mapping for teat number in an Iberian-by-Meishan pig intercross. Anim Genet 36: 490–496. 16293122 10.1111/j.1365-2052.2005.01358.x

[17] Noguera JL, Rodríguez C, Varona L, Tomàs A, Muñoz G, et al (2009) A bi-dimensional genome scan for prolificacy traits in pigs shows the existence of multiple epistatic QTL. BMC Genomics 10: 636. 20040109 10.1186/1471-2164-10-636 PMC2812473

[18] Fleige S, Pfaffl MW (2006) RNA integrity and the effect on the real-time qRT-PCR performance. Mol Aspects Med 27: 126–139. 16469371 10.1016/j.mam.2005.12.003

[19] Zhang X, Ding L, Sandford A (2005) Selection of reference genes for gene expression studies in human neutrophils by real-time PCR. BMC Mol Biol 6: 4. 15720708 10.1186/1471-2199-6-4 PMC551605

[20] Livak KJ, Schmittgen TD (2001) Analysis of relative gene expression data using real-time quantitative PCR and the 2-[delta][delta] CT method. Methods 25: 402–408. 11846609 10.1006/meth.2001.1262

[21] Gelfand AE, Smith AFM (1990) Sampling-based approaches to calculating marginal densities. J Am Stat Assoc 85: 398–409.

[22] Raftery AE, Lewis SM (1992) How many iterations in the gibbs sampler? In: Bernardo JM, Berger JO, David AP, Smith AFM, editors. Bayesian Statistics IV. Oxford, UK: Oxford University Press. 763–774.

