# Supplementary figures and images for: Selection of Internal Control Genes for Real-Time Quantitative PCR in Ovary and Uterus of Sows across Pregnancy
Source: PLoS One. 2013 Jun 13;8(6):e66023. doi: 10.1371/journal.pone.0066023 (PMC3681925; doi:10.1371/journal.pone.0066023)

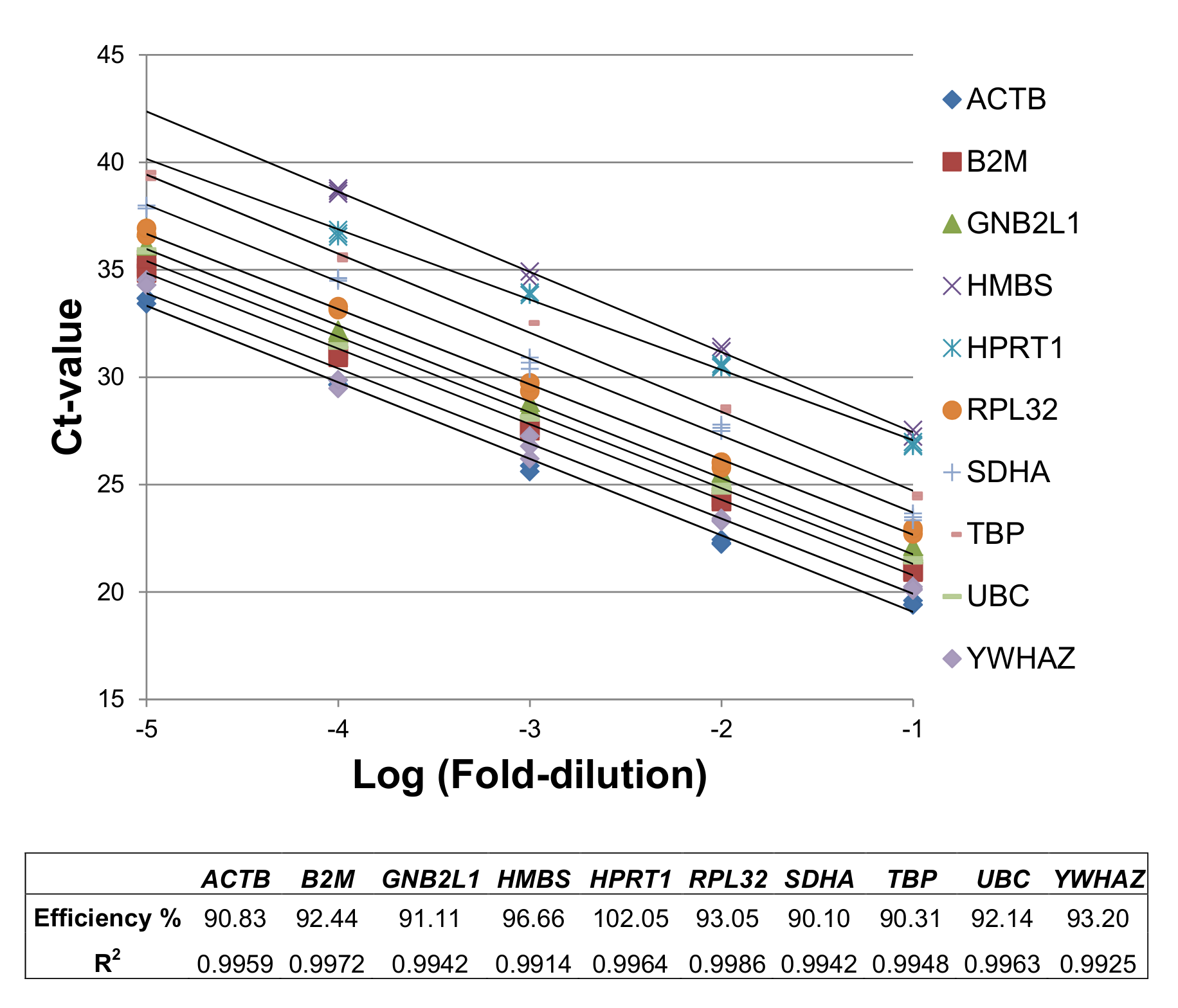

Supplement: Figure S1 — Linearity test and estimation of amplification efficiency for the PCR reaction of the ten candidate reference genes tested. (TIF) [file pone.0066023.s001.tif]
