# Supplementary material for: Selection of Internal Control Genes for Real-Time Quantitative PCR in Ovary and Uterus of Sows across Pregnancy
Source: PLoS One. 2013 Jun 13;8(6):e66023. doi: 10.1371/journal.pone.0066023 (PMC3681925; doi:10.1371/journal.pone.0066023)
Supplement: Table S1 — Intra-group gene expression variation for each individual gene in each different reproductive stage for each tissue, calculated by NormFinder . (DOCX) [file pone.0066023.s002.docx]

**Table S1.** Intra-group gene expression variation for each individual gene in each reproductive stage per tissue tissue, calculated by *NormFinder*.

|  | **Uterus** | | | | | **Ovary** | | | | |
| --- | --- | --- | --- | --- | --- | --- | --- | --- | --- | --- |
|  | **Heat** | **15 d** | **30 d** | **45 d** | **60 d** | **Heat** | **15 d** | **30 d** | **45 d** | **60 d** |
| *ACTB* | 0.377 | 0.635 | 0.215 | 0.540 | 0.163 | 1.422 | 0.053 | 0.117 | 0.133 | 0.129 |
| *B2M* | 0.515 | 3.141 | 0.099 | 0.852 | 0.143 | 0.083 | 0.040 | 0.057 | 0.032 | 0.263 |
| *GNB2L1* | 0.271 | 0.177 | 0.009 | 0.116 | 0.001 | 0.064 | 0.060 | 0.122 | 0.034 | 0.348 |
| *HMBS* | 0.146 | 0.620 | 1.779 | 0.950 | 0.075 | 0.470 | 0.012 | 0.036 | 0.036 | 0.006 |
| *HPRT1* | 0.033 | 0.984 | 0.005 | 0.002 | 0.067 | 0.034 | 0.119 | 0.039 | 0.006 | 0.079 |
| *RPL32* | 0.135 | 0.284 | 0.016 | 0.151 | 0.224 | 0.066 | 0.008 | 0.175 | 0.049 | 0.332 |
| *SDHA* | 0.216 | 0.045 | 0.007 | 0.026 | 0.026 | 0.077 | 0.071 | 0.110 | 0.067 | 0.250 |
| *TBP* | 0.161 | 0.007 | 0.038 | 0.009 | 0.024 | 0.020 | 0.016 | 0.023 | 0.021 | 0.097 |
| *UBC* | 0.020 | 0.019 | 0.007 | 0.004 | 0.001 | 0.107 | 0.026 | 0.056 | 0.003 | 0.077 |
| *YWHAZ* | 0.833 | 0.117 | 0.505 | 0.006 | 0.050 | 0.067 | 0.001 | 0.087 | 0.011 | 0.123 |
