# Supplementary material for: Selection of Internal Control Genes for Real-Time Quantitative PCR in Ovary and Uterus of Sows across Pregnancy
Source: PLoS One. 2013 Jun 13;8(6):e66023. doi: 10.1371/journal.pone.0066023 (PMC3681925; doi:10.1371/journal.pone.0066023)
Supplement: Table S2 — Pearson correlation coefficient (rI) between expression values of the ten reference gene in each tissue. (DOCX) [file pone.0066023.s003.docx]

**Table S2.** Pearson correlation coefficient (r_I_) between expression values of the ten reference gene in each tissue.

|  | **Uterus** | | | | | | | | |
| --- | --- | --- | --- | --- | --- | --- | --- | --- | --- |
| vs. | *ACTB* | *B2M* | *UBC* | *TBP* | *YWHAZ* | *GNB2L1* | *HMBS* | *HPRT* | *RPL32* |
| *B2M* | 0.375 | - | - | - | - | - | - | - | - |
| *UBC* | 0.755 | 0.458 | - | - | - | - | - | - | - |
| *TBP* | 0.820 | 0.521 | 0.919 | - | - | - | - | - | - |
| *YWHAZ* | 0.667 | 0.450 | 0.865 | 0.912 | - | - | - | - | - |
| *GNB2L1* | 0.920 | 0.366 | 0.785 | 0.859 | 0.752 | - | - | - | - |
| *HMBS* | 0.188 | 0.084 | 0.468 | 0.392 | 0.423 | 0.284 | - | - | - |
| *HPRT1* | 0.572 | 0.403 | 0.621 | 0.762 | 0.765 | 0.680 | 0.354 | - | - |
| *RPL32* | 0.787 | 0.362 | 0.830 | 0.914 | 0.861 | 0.899 | 0.282 | 0.741 | - |
| *SDHA* | 0.795 | 0.419 | 0.860 | 0.835 | 0.780 | 0.803 | 0.573 | 0.574 | 0.711 |
|  | **Ovary** | | | | | | | | |
| vs. | *ACTB* | *B2M* | *UBC* | *TBP* | *YWHAZ* | *GNB2L1* | *HMBS* | *HPRT* | *RPL32* |
| *B2M* | 0.788 | - | - | - | - | - | - | - |  |
| *UBC* | 0.805 | 0.789 | - | - | - | - | - | - |  |
| *TBP* | 0.946 | 0.803 | 0.827 | - | - | - | - | - |  |
| *YWHAZ* | 0.941 | 0.856 | 0.871 | 0.980 | - | - | - | - |  |
| *GNB2L1* | 0.868 | 0.676 | 0.742 | 0.936 | 0.908 | - | - | - |  |
| *HMBS* | 0.787 | 0.698 | 0.796 | 0.858 | 0.868 | 0.805 | - | - |  |
| *HPRT1* | 0.904 | 0.861 | 0.835 | 0.925 | 0.933 | 0.863 | 0.800 | - |  |
| *RPL32* | 0.861 | 0.677 | 0.739 | 0.934 | 0.905 | 0.993 | 0.817 | 0.849 |  |
| *SDHA* | 0.836 | 0.934 | 0.838 | 0.818 | 0.864 | 0.644 | 0.762 | 0.866 | 0.650 |
